# Development and validation of nomogram for predicting pathological complete response to neoadjuvant chemotherapy and immunotherapy for locally advanced gastric cancer: a multicenter real-world study in China

**DOI:** 10.3389/fimmu.2025.1603196

**Published:** 2025-07-18

**Authors:** Hao Cui, Rui Li, Liqiang Song, Yongpu Yang, Zhen Yuan, Xin Zhou, Junfeng Du, Chaojun Zhang, Hong Xu, Lin Chen, Yan Shi, Jianxin Cui, Bo Wei

**Affiliations:** ^1^ School of Medicine, Nankai University, Tianjin, China; ^2^ Department of General Surgery, The First Medical Center, Chinese PLA General Hospital, Beijing, China; ^3^ Department of Gastrointestinal Surgery, Peking University International Hospital, Beijing, China; ^4^ Department of General Surgery, Centre for Minimally Invasive Gastrointestinal Surgery, Southwest Hospital, Third Military Medical University, Chongqing, China; ^5^ Department of General Surgery, Peking University Third Hospital, Beijing, China; ^6^ Department of General Surgery, The Seventh Medical Center, Chinese PLA General Hospital, Beijing, China; ^7^ Department of General Surgery, The Sixth Medical Center, Chinese PLA General Hospital, Beijing, China; ^8^ Department of General Surgery, The Third Medical Center, Chinese PLA General Hospital, Beijing, China

**Keywords:** gastric cancer, pathological complete response, neoadjuvant, immunotherapy, nomogram

## Abstract

**Background:**

The combination of neoadjuvant chemotherapy and immunotherapy (NICT) brings a higher proportion of pathological complete response (pCR) compared with neoadjuvant chemotherapy for locally advanced gastric cancer (LAGC). Here we constructed and validated a prediction model to provide a clinical reference for predicting pCR.

**Methods:**

456 patients who accepted radical gastrectomy after NICT in seven large-scale gastrointestinal medical centers from Jan 2020 to Jan 2025 were enrolled in this study, with 320 patients in the training set and 136 patients in the validation set. The uni- and multivariate logistic regression model were used to evaluate the factors influencing pCR and a nomogram model was constructed. The area under the receiver operating characteristic curve (AUC), the calibration curve and decision curve analysis (DCA) were used to evaluate the discrimination, accuracy and clinical value of the nomogram model.

**Results:**

There was no significant difference in the baseline characteristics between training and validation set. The pCR and MPR rates were respectively 16.2% and 39.5%. Complete response by abdominal enhanced CT, less diameter of tumor bed, non-signet-ring cell, ages≥70 years old, and CEA<4.25 ng/mL were proved as the independent predictors for pCR (P<0.05). The nomogram model showed that the AUC (95%CI) predicting the pCR were 0.862 (95% CI: 0.807-0.916) in the training set and 0.934(95%CI: 0.889-0.979) in the validation set. The calibration curves showed that the prediction curve of the nomogram was good in fit with the actual pCR in the training and validation set respectively (Hosmer-Lemeshow test: χ2 = 9.093, P=0.168; χ2 = 2.853, P=0.827). Decision curve analysis showed a good outcome to assess net benefit.

**Conclusion:**

Our nomogram model could provide satisfactory predictive effect for the pCR in LAGC patients with NICT, which proves to be a valuable approach for surgeons to make personalized strategies.

## 1 Introduction

According to the latest data from Global Cancer Statistics 2020, gastric cancer (GC) ranks as the fifth most common malignancy and the fourth leading cause of cancer-related mortality worldwide (1). In China, GC remains the third most prevalent cause of cancer-related deaths, representing a substantial public health burden (2). Despite the gradual implementation of early cancer screening programs in China, a significant proportion of GC patients are still diagnosed at advanced stages, largely due to unfavorable dietary habits, high rates of Helicobacter pylori infection, smoking, and other risk factors (3). A comprehensive periopreative treatment approach has become a crucial strategy to enhance the efficacy for advanced gastric cancer (AGC).

For unresectable AGC, immunotherapy, particularly immune checkpoint inhibitors (ICIs), has demonstrated significant improvements in overall survival when combined with chemotherapy compared to chemotherapy alone (4, 5). When mentioned to the neoadjuvant therapy phase, immunotherapy has shown the potential to amplify and transcriptionally modify tumor-specific T cell clones, thereby enhancing both local and systemic anti-tumor immune responses (6). Consequently, there has been growing interest among clinicians in the therapeutic potential of neoadjuvant chemotherapy combined with immunotherapy (NICT) for locally advanced gastric cancer (LAGC).

In 2024, a phase II study from China suggesting that neoadjuvant toripalimab plus SOX or XELOX significantly improved pathological complete response (pCR) compared to neoadjuvant chemotherapy alone (22.2% vs.7.4%, P=0.030) (7), Furthermore, findings from the DANTE study and the KEYNOTE-585 trial also suggest that NICT leads to superior tumor downstaging (8, 9).

Tumor regression grade (TRG) is a critical metric for evaluating pathological response to neoadjuvant therapy. Within the Becker criteria framework, major pathological response (MPR), defined as TRG1a/1b, and pCR, defined as TRG1a, are widely used endpoints in clinical studies (10). Numerous studies have demonstrated that TRG is strongly associated with overall survival (OS), and patients achieving a favorable pathological response are likely to experience significant survival benefits in LAGC (11, 12), Therefore, accurately predicting pathological response to neoadjuvant therapy is essential for identifying sensitive populations and optimizing treatment strategies.

Recent efforts have increasingly focused on predicting pathological response to neoadjuvant therapy using preoperative laboratory indicators, radiomics, immune microenvironment analysis, and other advanced approaches (13, 14). However, there remains a lack of predictive models specifically designed to assess pathological efficacy in LAGC patients undergoing NICT. Here, we conducted a multicenter retrospective study to develop a nomogram incorporating preoperative radiological response, laboratory indicators, and pathological characteristics to predict pCR in LAGC patients who underwent gastrectomy following NICT. This model aims to provide a valuable tool for identifying sensitive populations and guiding personalized treatment strategies.

## 2 Materials and methods

### 2.1 Study population

456 LAGC patients who accepted gastrectomy plus D2 lymphadenectomy after NICT in the seven large-scale gastrointestinal centers between Jan 2020 and Jan 2025 were included into this study. They were randomly allocated into the training cohort and the validation cohort at a ratio of 7:3 (training cohort: n=320, validation cohort: n=136). The inclusion criteria were as follows: (a) Clinical stage II-IVa according to the American Joint Committee on Cancer (AJCC) 8th edition gastric cancer staging criteria; (b) Pathologically confirmed as gastric adenocarcinoma; (c) ASA grade≤III; (d) HER-2 negativity confirmed by immunohistochemical method and fluorescence *in situ* hybridization (FISH) test; (e) received gastrectomy combined with D2 lymphadenectomy after treatment;(f) availability of complete clinical-pathological data. Exclusion criteria were as follows: (a) distant metastasis before treatment; (b) prior chemotherapy, targeted therapy, immunotherapy, or radiotherapy; (c) recurrent gastric cancer; (d) concurrent malignancies. This study was approved by the Ethic Committee of the Chinese PLA general hospital (No. S2022-299-01).

### 2.2 Treatment

Patients in this study received preoperative concurrent chemotherapy and immunotherapy. The neoadjuvant chemotherapy regimens included dual-agent protocols—SOX (S-1 + oxaliplatin), XELOX (capecitabine + oxaliplatin), SAP (S-1 + nab-paclitaxel), and FOLFOX (oxaliplatin + fluorouracil)—as well as the triple-agent FLOT regimen (fluorouracil + oxaliplatin + docetaxel). Neoadjuvant immunotherapy regimens were administered as programmed death-1 (PD-1) inhibitors, which included nivolumab, pembrolizumab, toripalimab, camrelizumab, and sintilimab. Treatment cycles ranged from 2 to 8, with all patients completing combined therapy prior to surgery. None of the patients in the study received dual immunotherapy before surgery. Radical gastrectomy was performed 4–6 weeks after the final NICT cycle. During NICT, the treatment-related adverse events (TRAEs) were categorized and graded according to the Common Terminology Criteria for Adverse Events criteria (version 5.0) (15).

In addition, in terms of the effectiveness of neoadjuvant therapy, radiological responses were assessed using the Response Evaluation Criteria in Solid Tumors criteria (version 1.1), with classifications including complete response (CR), partial response (PR), stable disease (SD), and progressive disease (PD) (16). According to the 8th edition of the AJCC Cancer Staging Manual (17), the post-neoadjuvant therapy pathological TNM staging was defined as ypTNM, which stratifies non-metastatic locally advanced gastric cancer (LAGC) patients following neoadjuvant therapy into Stage I, II, and III. The ypT and ypN classifications were determined by the deepest residual tumor infiltration within the gastric wall and regional lymph node metastasis status, respectively.

### 2.3 Pathological response

For pathological evaluation, Resected specimens were independently reviewed by two senior pathologists. We used Becker classification to evaluate TRG (10). TRG was classified as follows: TRG1a: no residual tumor; TRG1b: less than 10% residual tumor; TRG2: 10%-50% residual tumor; TRG3: over 50% residual tumor. Pathological complete response (pCR) and major pathological response (MPR) were respectively defined as TRG1a and TRG1a/1b.

### 2.4 Statistical analysis

We used SPSS 26.0(IBM SPSS Statistics, Chicago, IL, USA), and R version 4.2.2 (R-Project, Institute of Statistics and Mathematics, Vienna, Austria) to perform statistical analysis. Chi-square χ^2^ test and Fisher’s exact test were used to analyze the differences in categorical variables between training cohort and external validation cohort, while the Wilcoxon rank test or Student’s *t*-test was used for continuous variables. Univariate logistic regression identified predictors of pCR(P<0.05), which were subsequently incorporated into multivariate logistic models. The cut-off values of the CEA was selected using the Youden Index from the receiver operating characteristic (ROC) curve and clinical preference. The nomogram was constructed using independent predictors from the training cohort with the rms package (Version: 6.2-0) in R software. Subsequently, the performance of the logistic regression model was quantified by evaluating discrimination and calibration in both the training and validation cohorts. The concordance index (C-index), which measures the agreement between predicted probabilities and actual outcomes, was calculated to assess the prediction and discrimination ability of the model. Calibration curves were generated using 1000 bootstrap replicates to validate the calibration of the nomogram. The Hosmer-Lemeshow test was applied to evaluate the fitness of the model. Additionally, decision curve analysis (DCA) was conducted to assess the clinical utility of the nomogram. P <0.05 was defined as a statistically significant difference.

## 3 Results

### 3.1 Baseline characteristics between training and validation set

The baseline characteristics of patients in the training set (n=320) and validation set (n=136) are summarized in **Table 1**. In terms of demographic and clinical variables, no significant difference was observed in sex distribution (male: 79.7% vs. 72.8%, P=0.106), age (61.58 ± 9.67 vs. 61.96 ± 9.64 years, P=0.700), BMI (23.71 ± 3.33 vs. 23.38 ± 3.01 kg/m², P=0.327), or treatment cycles (≤4 cycles: 75.0% vs. 79.4%, P=0.311). Similarly, immunotherapy regimens (domestic drug: 83.4% vs. 79.4%, P=0.303) and chemotherapy regimens (SOX/XELOX: 91.3% vs. 86.0%, P=0.093) demonstrated comparable distributions between the two cohorts.

**Table 1:** Baseline characteristics between training set and validation set.

Characteristics	Training set (n=320)	Validation set (n=136)	Statistic value	P value
Sex, n (%)			2.612	0.106
Male	255 (79.7)	99 (72.8)		
Female	65 (20.3)	37 (27.2)		
Ages (x ± s, y)	61.58 ± 9.67	61.96 ± 9.64	-0.376	0.700
BMI, x ± s (kg/m^2^)	23.71 ± 3.33	23.38 ± 3.01	0.981	0.327
Treatment cycles, n (%)			1.028	0.311
≤4	240 (75.0)	108 (79.4)		
>4	80 (25.0)	28 (20.6)		
Immunotherapy regimens, n (%)			1.059	0.303
Domestic drug	267 (83.4)	108 (79.4)		
Import drug	53 (16.6)	28 (20.6)		
Chemotherapy regimens, n (%)			2.814	0.093
SOX/XELOX	292 (91.3)	117 (86.0)		
Others	28 (8.8)	19 (14.0)		
Severe TRAEs, n (%)			2.762	0.097
No	266 (83.1)	104 (76.5)		
Yes	54 (16.9)	32 (23.5)		
Radiological response, n (%)			-1.057	0.290
CR	25 (7.8)	11 (8.1)		
PR	177 (55.3)	64 (47.1)		
SD	103 (32.2)	59 (43.4)		
PD	15 (4.7)	2 (1.5)		
Tumor location, n (%)			5.712	0.126
Proximal 1/3	159 (49.7)	66 (48.5)		
Middle 1/3	62 (19.4)	19 (14.0)		
Distal 1/3	87 (27.2)	49 (36.0)		
Diffused	12 (3.8)	2 (1.5)		
cTNM stage, n (%)			-0.444	0.657
II	16 (5.0)	9 (6.6)		
III	288 (90.0)	120 (88.2)		
IVa	16 (5.0)	7 (5.1)		
ypT, n (%)			44.492	<0.001
0	56 (17.5)	24 (17.6)		
1	63 (19.7)	18 (13.2)		
2	35 (10.9)	14 (10.3)		
3	139 (43.4)	36 (26.5)		
4	27 (8.4)	44 (32.4)		
ypN, n (%)			6.888	0.076
0	167 (52.2)	74 (54.4)		
1	52 (16.3)	33 (24.3)		
2	45 (14.1)	13 (9.6)		
3	56 (17.5)	16 (11.8)		
ypTNM stage, n (%)			-0.713	0.476
0	50 (15.6)	24 (17.6)		
I	89 (27.8)	23 (16.9)		
II	77 (24.1)	44 (32.4)		
III	104 (32.5)	45 (33.1)		
Tumor differentiation, n (%)			1.768	0.184
Well/Moderate	184 (57.5)	69 (50.7)		
Poor/Undifferentiated	136 (42.5)	67 (49.3)		
Diameter of tumor bed, x ± s (cm)	3.57 ± 2.06	3.53 ± 2.31	0.192	0.848
Signet-ring cell carcinoma, n (%)			0.144	0.705
No	249 (77.8)	108 (79.4)		
Yes	71 (22.2)	28 (20.6)		
Vascular invasion, n (%)			6.481	0.011
No	240 (75.0)	86 (63.2)		
Yes	80 (25.0)	50 (36.8)		
Nerve invasion, n (%)			7.515	0.006
No	228 (71.3)	79 (58.1)		
Yes	92 (28.8)	57 (41.9)		
TRG grade, n (%)			-3.505	<0.001
Ia	50 (15.6)	24 (17.6)		
Ib	89 (27.8)	17 (12.5)		
II	122 (38.1)	41 (30.1)		
III	59 (18.4)	54 (39.7)		
Pathological complete response, n (%)	50 (15.6)	24 (17.6)	0.287	0.592
Major pathological response, n (%)	139 (43.4)	41 (30.1)	7.056	0.008

BMI, body mass index; TRAEs, treatment-related adverse events; CR, complete response; PR, partial response; SD, stable disease; PD, progressive disease; TRG, tumor regression grade.

Pathological and treatment-related features also showed balanced profiles, including radiological response (CR: 7.8% vs. 8.1%, P=0.290), tumor location (proximal 1/3: 49.7% vs. 48.5%, P=0.126), diameter of tumor bed (3.57 ± 2.06 vs. 3.53 ± 2.31 cm, P=0.848), signet-ring cell carcinoma (22.2% vs. 20.6%, P=0.705), cTNM stage (III: 90.0% vs. 88.2%, P=0.657), and tumor differentiation (well/moderate: 57.5% vs. 50.7%, P=0.184). However, significant disparities were identified in vascular invasion (25.0% vs. 36.8%, P=0.011), nerve invasion (28.8% vs. 41.9%, P=0.006), TRG grade (Grade III: 18.4% vs. 39.7%, P<0.001), and ypT stage (ypT4: 32.4% vs. 8.4%, P<0.001).

Overall, among the 456 patients, 74 (16.2%) patients acquired pCR, while 180 (39.5%) patients acquired MPR. No significant difference in the analyzed factors was observed between the training and validation cohorts. Further characterization of the 74 pCR patients, including demographic and tumor-specific features, is provided in **Supplementary Table 1**.

### 3.2 Univariate and multivariate logistic analysis of training set

The univariate - and multivariate logistic regression results used to explore the independent factors for pathological complete response after NICT in the training set are shown in **Table 2**. We placed indicators obtained by the univariable logistic regression with a p-value of < 0.05 into the multivariable analysis and observed that age ≥70 years (odds ratio [OR]: 3.030, 95% confidence interval [CI]: 1.327–6.918, P = 0.009), less diameter of tumor bed (OR: 0.613, 95% CI: 0.469–0.801, P < 0.001), Complete response by abdominal enhanced CT(OR: 0.092, 95% CI: 0.033–0.257, P < 0.001), non-signet-ring cell carcinoma (OR: 0.108, 95% CI: 0.014–0.838, P = 0.033), CEA after NICT<4.25 ng/mL(OR: 0.351, 95% CI: 0.136–0.908, P = 0.029) were significant independent predictors for pCR.

**Table 2:** Uni- and multivariate logistic regression analysis for pathological complete response after NICT in the training set.

Factor	Univariate analysis		P value	Multivariate analysis		P value
	OR	95%CI		OR	95%CI	
Sex			0.056			
Male	1.000					
Female	0.389	0.148-1.023				
Age (y)			0.023			0.009
<70	1.000			1.000		
≥70	2.176	1.112-4.258		3.030	1.327-6.918	
Tumor location			0.571			
Upper 1/3	1.000					
Others	1.191	0.651-2.182				
Radiological response			<0.001			<0.001
CR	1.000			1.000		
Non-CR	0.059	0.024-0.148		0.092	0.033-0.257	
Treatment cycles			0.859			
≤4	1.000					
>4	0.938	0.464-1.898				
Severe TRAEs			0.555			
No	1.000					
Yes	0.772	0.327-1.823				
NRS-2002 score			0.583			
<3	1.000					
≥3	1.184	0.647-2.166				
Diameter of tumor bed(cm)	0.570	0.451-0.721	<0.001	0.613	0.469-0.801	<0.001
Tumor differentiation			0.586			
Well/Moderate	1.000					
Poor/Undifferentiation	1.184	0.645-2.170				
Signet-ring cell carcinoma			0.005			0.033
No	1.000			1.000		
Yes	0.058	0.008-0.430		0.108	0.014-0.838	
PNI score			0.138			
<45	1.000					
≥45	0.607	0.314-1.175				
NLR			0.151			
<2.6	1.000					
≥2.6	0.555	0.248-1.240				
PLR			0.209			
<105	1.000					
≥105	0.677	0.369-1.243				
CEA after NICT			0.003			0.029
<4.25	1.000			1.000		
≥4.25	0.255	0.105-0.621		0.351	0.136-0.908	

TRAEs, treatment-related adverse events; NRS-2002, nutritional risk screening 2002; PNI, prognostic nutritional index; NLR, neutrophil-to-lymphocyte ratio; PLR, platelet-to-lymphocyte ratio; CEA, carcinoembryonic antigen; NICT, neoadjuvant chemotherapy and immunotherapy.

### 3.3 Nomogram for predicting pCR for LAGC patients with NICT

**Figure 1:**
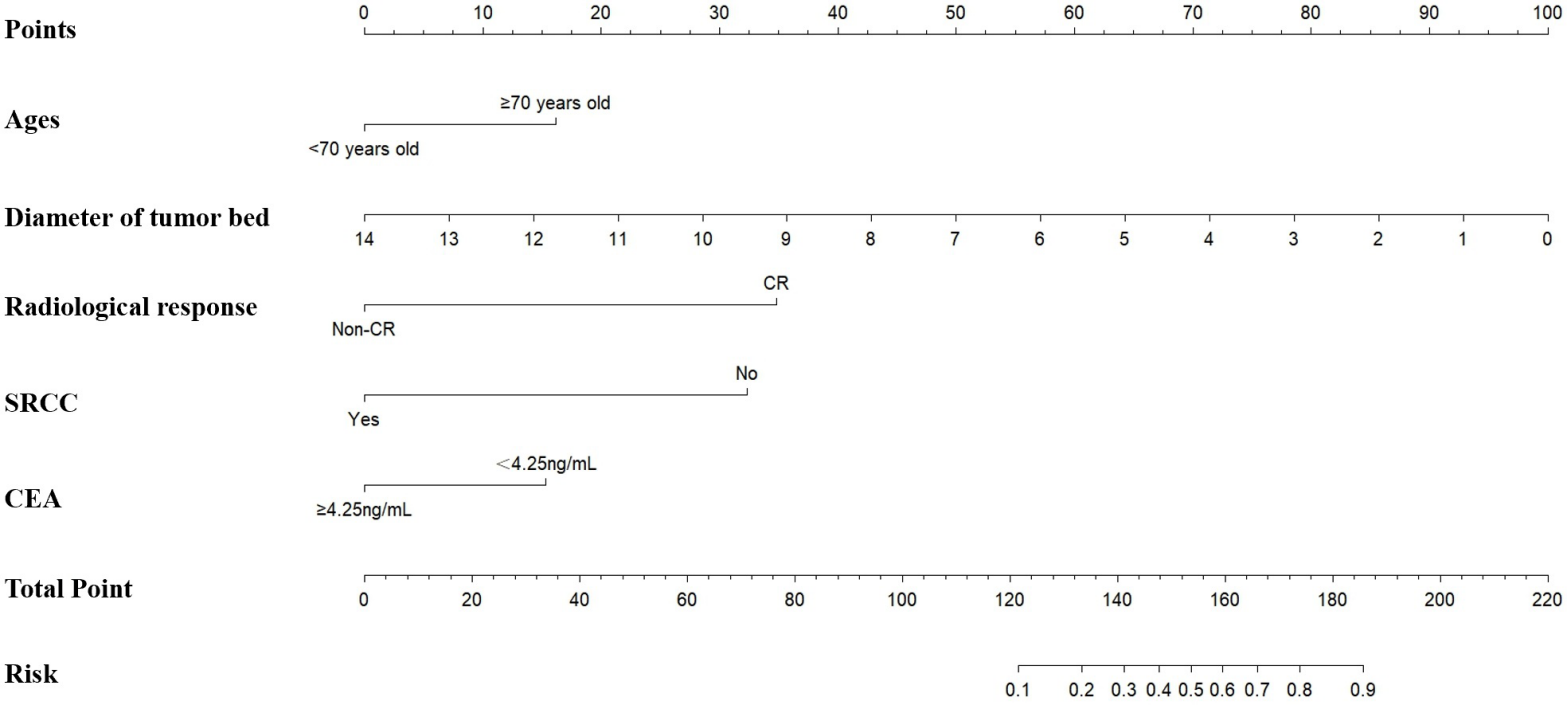
The training set based nomogram for predicting the probability of pCR after NICT in LAGC patients. The vertical axis displays various variables affecting pCR, including age, diameter of the tumor bed, radiological response, SRCC, and CEA. The upper horizontal axis represents the points corresponding to each variable, ranging from 0 to 100. The lower horizontal axis indicates the total points, ranging from 0 to 220, and the associated risk value, ranging from 0.1 to 0.9. pCR, pathologic complete response; NICT, neoadjuvant chemotherapy and immunotherapy; LAGC, locally advanced gastric cancer; SRCC, signet-ring cell carcinoma.

Based on the multivariate logistic regression analysis of the training cohort (n=320), five independent predictors of pathological complete response (pCR) were identified: Complete response by abdominal enhanced CT, less diameter of tumor bed, non-signet-ring cell carcinoma, ages≥70 years old, and CEA<4.25 ng/mL. These variables were incorporated into a nomogram to predict the probability of pCR in locally advanced gastric cancer (LAGC) patients receiving neoadjuvant chemotherapy combined with immunotherapy (NICT) (**Figure 1**).

Each predictor was assigned a weighted score proportional to its regression coefficient in the multivariate model. The total score, calculated by summing individual scores, was mapped to a predicted probability of pCR on a scale of 10%-90%. This visual tool allows clinicians to estimate personalized pCR probabilities by integrating preoperative radiological response, laboratory indicators, and pathological characteristics.

### 3.4 Establishment and verification of the nomogram multi−factor prediction model

**Figure 2:**
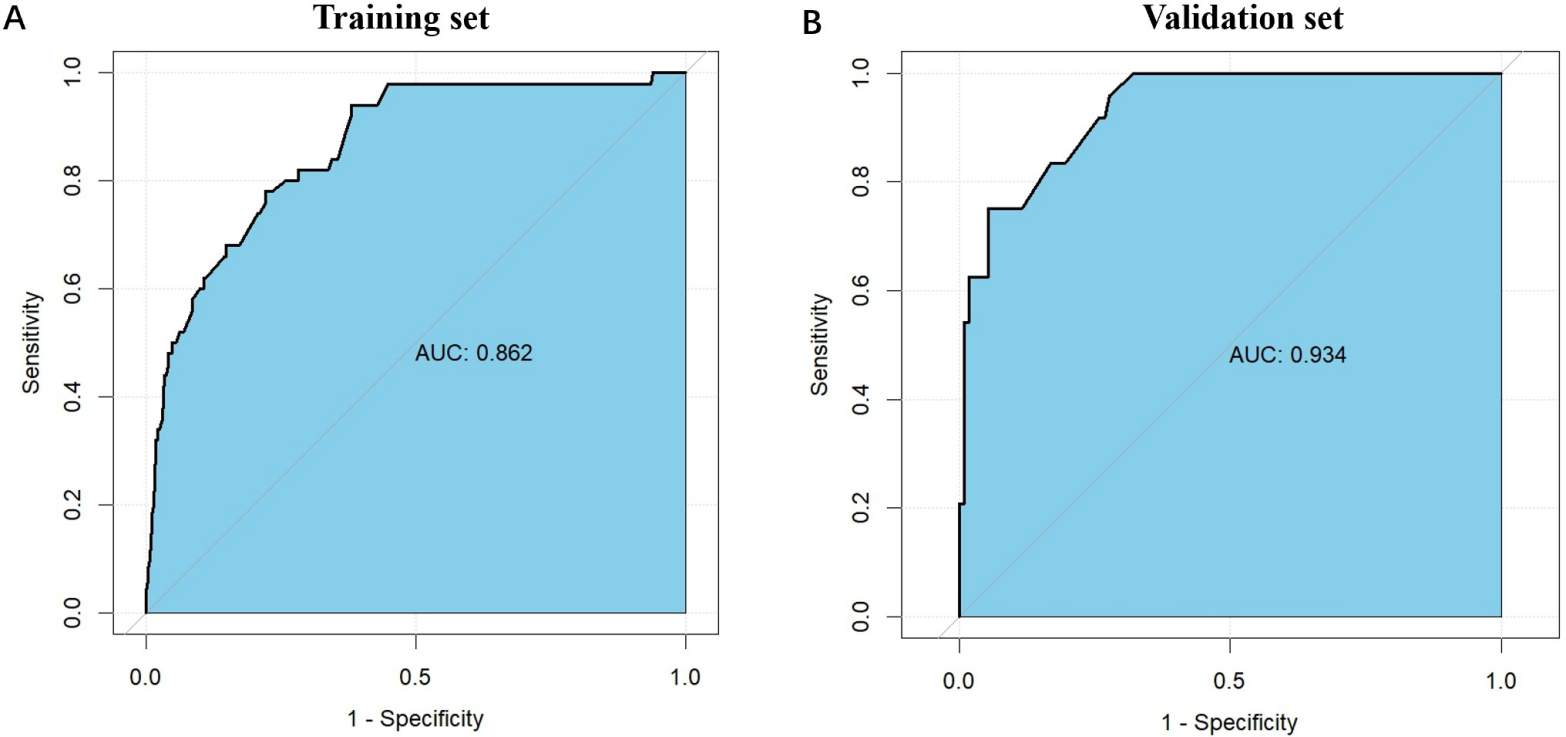
The ROC curves for the nomogram model in the training cohort and validation cohort. **(A)** The ROC curves for the nomogram model in the training cohort; **(B)** The ROC curves for the nomogram model in the validation cohort. The horizontal axis represents 1 - Specificity (false positive rate), while the vertical axis denotes Sensitivity (true positive rate). The curve demonstrates the trade-off between sensitivity and 1 - specificity at different thresholds, with curves closer to the upper left corner indicating better model performance. The AUC values for the training and validation sets are 0.862 and 0.934, respectively, suggesting slightly better performance on the validation set and good generalization ability of the model. ROC, receiver operating characteristic.

The nomogram demonstrated robust predictive performance in both the training and validation cohorts. In the training set, the area under the receiver operating characteristic curve (AUC) was 0.862 (95% CI: 0.807–0.916), indicating excellent discrimination (**Figure 2A**). External validation using the validation set (n=136) yielded an even higher AUC of 0.934 (95% CI: 0.889–0.979) (**Figure 2B**), confirming generalizability of the model.

Subgroup analyses were conducted to evaluate the predictive performance of the nomogram across different clinical features. Binary variables, including tumor location, gender, and tumor differentiation, were selected for subgroup analysis due to the limited sample size. The nomogram demonstrated consistent predictive accuracy across all subgroups in the training set, validation set, and overall population, with AUC values exceeding 0.800 in all subgroups. This consistency underscores the clinical applicability of the prediction model (**Supplementary Figure 1**).

**Figure 3:**
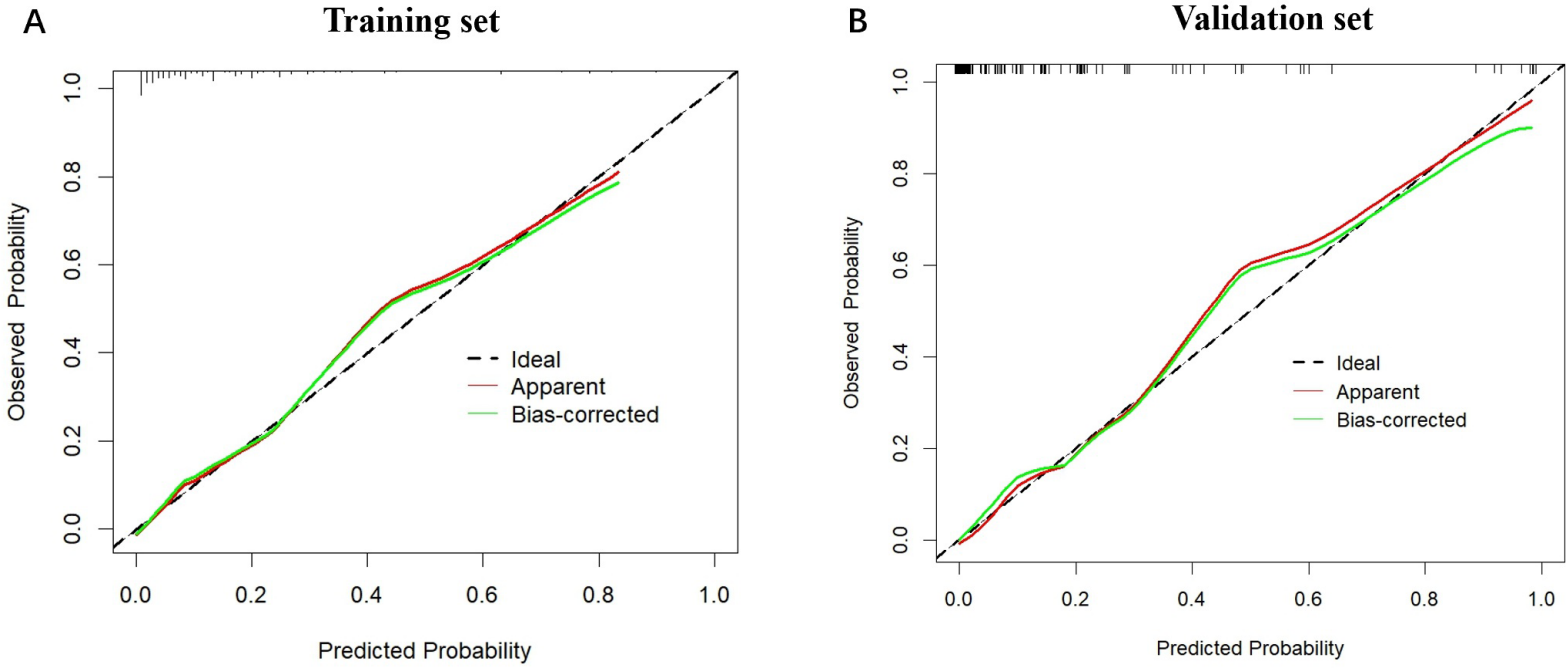
The calibration plots for the nomogram model in the training cohort and validation cohort. **(A)** The calibration plots for the nomogram model in the training cohort; **(B)** The calibration plots for the nomogram model in the validation cohort. The horizontal axis represents the predicted probability, while the vertical axis shows the observed probability. The red solid line represents the apparent calibration curve without bias correction, while the green solid line shows the bias-corrected curve, which is closer to the ideal line after correction.

**Figure 4:**
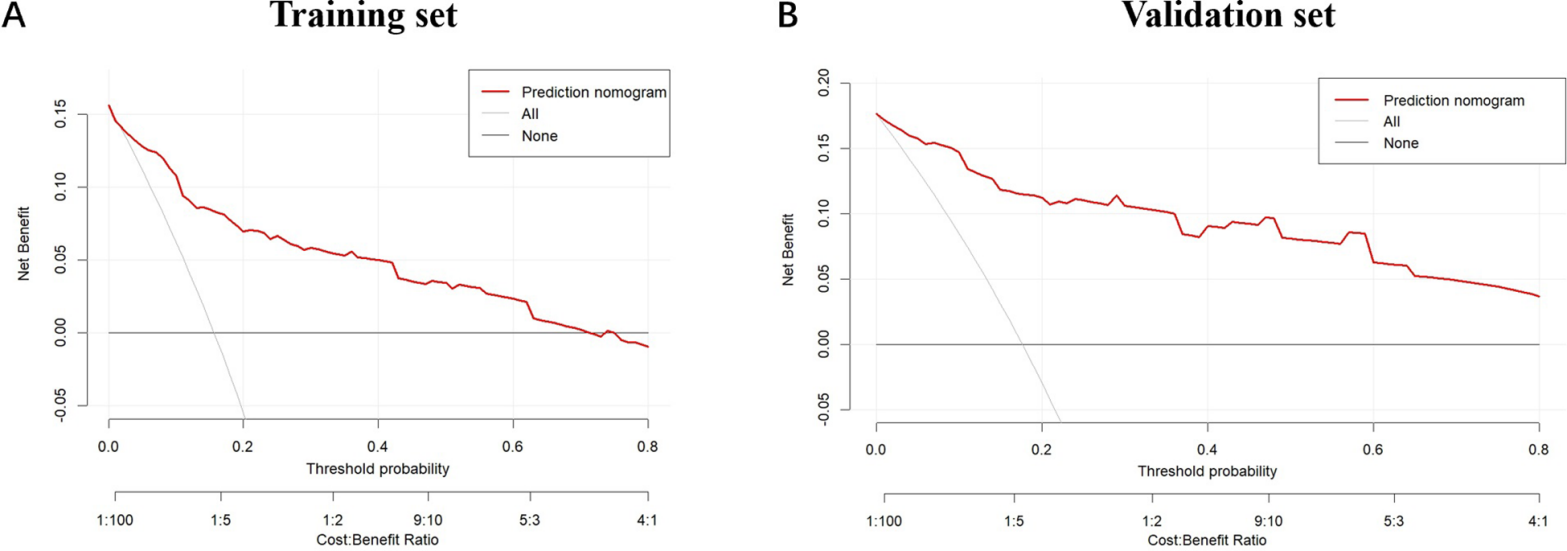
The decision curves for the nomogram in the training cohort and validation cohort. **(A)** The decision curves show the net-benefit of using the nomogram in the training cohort; **(B)** The decision curves show the net-benefit of using the nomogram in the validation cohort. The horizontal axis represents the threshold probability, and the vertical axis indicates the net benefit at different threshold probabilities. The red curve illustrates the net benefit of the prediction model at various threshold probabilities. The gray curve represents the net benefit assuming all samples are positive, and the black curve represents the net benefit assuming all samples are negative. This chart evaluates the practicality and economic efficiency of the model under different cost-benefit ratios.

Furthermore, ROC curve analysis of multiple indicators in the training set confirmed the superior diagnostic value of the multivariable nomogram compared to single-variable models. The nomogram exhibited higher AUC (95% CI) than individual variables, indicating enhanced predictive capability (**Supplementary Figure 2**).Calibration curves revealed strong agreement between predicted and observed pCR probabilities in both cohorts (**Figure 3**). The Hosmer-Lemeshow test showed no significant deviation from perfect fit (training set: χ²=9.093, P=0.168; validation set: χ²=2.853, P=0.827). Decision curve analysis (DCA) further validated the clinical utility of the nomogram, demonstrating a significant net benefit across a wide range of threshold probabilities (**Figure 4**).

## 4 Discussion

Numerous studies currently indicated that NICT can lead to improved pathological response, thereby positively impacting the long-term prognosis for LAGC patients. It is crucial to construct a prediction model for tumor regression so that provide reference for surgeons to choose the appropriate timing of surgery and identify sensitive populations.

Nomogram is a convenient prediction tool based on the results of the multivariate regression analysis. It has also been widely used for predicting the tumor response after neoadjuvant therapy. Ma et al. performed a nomogram to predict objective response after NICT for esophageal cancer (16). A nomogram based on creatinine-to-cystatin C ratio and body composition from Ji H’s study showed a good performance in predicting overall survival after PD-1 inhibitors-based combination treatment in metastatic GC (18). Similarly, Zhao et al. and Wang et al. applied nomograms in metastatic melanoma and elderly primary colorectal lymphoma, demonstrating accurate prognostic predictions by integrating baseline biomarkers and comprehensive clinical data, respectively (19, 20). In this study, we also developed a predictive nomogram utilizing the clinicopathological characteristics such as radiological evaluation, laboratory indicators, and preoperative gastroscopy to forecast the degree of pathological response in LAGC patients undergoing gastrectomy after NICT. Our findings revealed that the AUC (95%CI) of nomogram based on five indicators including radiological response, tumor bed diameter, signet-ring cell carcinoma, age, and CEA after NICT in the training dataset was 0.862 (95% CI:0.807-0.916), while the AUC (95%CI) in the validation dataset was 0.934(95%CI:0.889-0.979), which indicate the robust predictive accuracy and provide a reference for surgeons to make individual therapeutic strategies. The model can be further improved by external validation set verification and exploring more clinicopathological factors to optimize the clinical utility of the nomogram for guiding personalized therapeutic strategies in LAGC.

Signet-ring cell carcinoma (SRCC) is a highly malignant and invasive type of gastric cancer. SRCC has a poorer long-term prognosis compared to well to moderate differentiated adenocarcinoma for LAGC (21). The unique pathological type of SRCC might affect its response to anticancer therapy. Puccini A et al. showed that SRCC had a higher frequency of mutations in CDH1, BAP1, and ERBB2, compared to non-SRCC in the gastric cancer cohort (22). A systematic review performed that PD-L1 overexpression, NGS-MSI, and TMB was respectively observed in 45%, 3.5%, and 1.8% of SRCCs, all of which were lower than in non-SRCCs but did not achieve statistical significance (23). In this study, we found that patients with SRCC had significantly lower pCR rates than those without SRCC (1.0% vs. 20.4%, χ^2^ = 17.239, P<0.001). SRCC was the independent risk factor of non-pCR for LAGC patients receiving NICT (P=0.033), which demonstrated that SRCC might have poor response to NICT for LAGC. Chen et al. attributed this phenomenon to the lack of CD8-Tex derived CXCL13 and tertiary lymphoid structures (TLSs) (24). However, we still need to acknowledge the positive role of the clinicopathological and molecular characteristics such as dMMR, PD-L1 combined positive score (CPS) ≥ 5, and CDH1 wild type in improving the prognosis of SRCC patients (25).

CEA is one of the most common tumor biomarkers which has been widely used for tumor diagnosis, monitoring of recurrence, and evaluation of perioperative treatment. Tang XH et al. found that CEA was a strong marker with a favorable treatment response and survival benefits for LAGC patients who accepted neoadjuvant chemotherapy (26). Zwart WH’s study revealed that CEA could be a suitable target for response evaluation after neoadjuvant treatment for rectal cancer (27). A meta-analysis performed that >5 ​ng/mL pre-therapeutic serum concentration of CEA was significantly associated with tumor response (28). In this study, we found that LAGC patients with lower preoperative CEA level of<4.25 ng/mL had more probability to acquire pCR compared with those with higher CEA level. Preoperative lower CEA level can be considered as a promising biomarker for predicting better tumor response after NICT. Thus, it is necessary to focus on the preoperative CEA levels to evaluate the appropriate timing of surgery during NICT.

Multiple studies have demonstrated that smaller diameter of tumor bed correlates with favorable pathological response and improved prognosis (29, 30), which aligns with the predictive outcomes of our nomogram. We hypothesize that reduced tumor bed dimensions may indicate heightened sensitivity to neoadjuvant therapy. Based on these findings, we recommend the routine preoperative implementation of gastroscopy for tumor bed size assessment as a critical parameter in predicting neoadjuvant treatment efficacy. However, it should be noted that the tumor bed comprises not only residual viable tumor but also necrosis, inflammatory infiltrates and fibrotic alterations, factors that potentially compromise the precision of post-neoadjuvant therapeutic evaluations (31). Recent advances in artificial intelligence (AI) and machine learning in image recognition and analysis have demonstrated significant progress, holding promising potential for application in pathological evaluation (32, 33). These technologies may assist pathologists in alleviating the workload associated with tumor bed characterization, thereby enhancing the accuracy and efficiency of post-treatment assessment.

Radiological evaluation using the Response Evaluation Criteria in Solid Tumors (RECIST) was regarded as the standard approach to evaluate tumor response after perioperative therapy (18). In this study, we found that complete response (CR) using the RECIST v1.1 criteria was the independent predictive indicator for pCR with acceptable diagnostic capacity [AUC (95%CI): 0.862(0.807-0.916)], which presented the pivotal role of preoperative radiological assessment. However, it is still difficult to immediately distinguish the pseudo progression from true disease progression due to the reason of immune checkpoint inhibitors activate immune cells, resulting in a large accumulation of immune cells on the surface or inside of the primary lesion and small metastatic lesions (34). According to the previous studies, the incidence of pseudo progression was ranged from 0% to 15% (35). Thus, we still need to dynamically evaluate the radiological response and rely on the combined application of other clinical indicators to improve the exact predictive value of pCR in the LAGC patients after NICT.

An interesting finding in our study was that LAGC patients aged ≥70 years achieved a higher pCR rate following NICT compared to those aged <70 years (25% vs. 13.3%, χ^2^ = 5.333, P=0.021), which demonstrated the better tumor response for elderly LAGC patients. A study has found that the incidence of dMMR/MSI-H increases with age, indicating to some extent that elderly gastric cancer patients are more likely to benefit from neoadjuvant immunotherapy than young gastric cancer patients (36). Consistent with this, Erbe et al. demonstrated that the accumulation of tumor mutational burden (TMB), upregulation of immune checkpoint genes (e.g., PD-L1), and activation of interferon-gamma (INF-γ) signaling pathways in tumors of elderly patients may synergistically enhance the efficacy of immune checkpoint blockade (ICB) (37). In addition, Jiang et al. identified age as an independent risk factor influencing the therapeutic response to neoadjuvant chemotherapy in gastric cancer patients, with elderly patients exhibiting higher pathological response rates compared to younger individuals (38). Similarly, Rombouts et al. reported that age serves as an independent predictor of pCR in CRC, revealing that younger patients exhibited reduced pCR rates (39), attributable to the higher malignancy and aggressiveness of tumors in this population (40). Further studies should focus on the association between tumor response and deep mechanism for elderly LAGC patients following NICT.

In recent years, immunotherapy has progressively revolutionized the therapeutic landscape of gastric cancer, demonstrating a paradigm shift from third-line to first-line settings (4, 8, 9). Immunotherapy has now become a standard treatment for advanced gastric cancer, and its application in the perioperative setting is being actively explored in multiple countries (41, 42). The potential benefits of immunotherapy in the perioperative management of gastric cancer are likely mediated by several mechanisms: (1) Neoadjuvant immunotherapy reactivates tissue-resident memory CD8+ T cells, promoting their expansion and diversification (43, 44). (2) Following resection of the primary tumor, circulating tumor-specific CD8+ T cells persist, potentially enhancing T cell infiltration in residual micrometastatic sites and broadening the spectrum of tumor-specific T cell responses. (3) Preclinical studies have demonstrated that eradication of tumor cells can establish tumor-specific CD8+ T cell memory, conferring long-term survival advantages (44). Consequently, the integration of immunotherapy during perioperative treatment may yield clinical benefits for patients (45). As demonstrated in our meta-analysis, PD-1 inhibitor combined with neoadjuvant chemotherapy (NCT) significantly improves the likelihood of achieving radical surgery and prognosis in LAGC patients. Specifically, the NICT group exhibited significantly higher rates of pCR (P < 0.001) and R0 resection (P = 0.001), alongside a notably lower 2-year recurrence rate (P = 0.001) compared to the NCT group (46). At the American Society of Clinical Oncology Gastrointestinal Cancers Symposium (ASCO-GI), the pivotal phase III KEYNOTE-585 trial (9)—a randomized, double-blind study of neoadjuvant immunotherapy for gastric cancer—reported a statistically significant pCR improvement with pembrolizumab plus FLOT versus placebo plus FLOT (14% vs. 6.8%, P < 0.05). Subsequently, at the 2025 ASCO Annual Meeting, the MATTERHORN study (47)—the first phase III trial to demonstrate positive perioperative immunotherapy outcomes in gastric cancer—showed a significantly higher pCR rate with durvalumab plus FLOT compared to placebo plus FLOT (19% vs. 7%, P < 0.001). These landmark studies collectively demonstrate the efficacy of neoadjuvant immunotherapy combined with chemotherapy for LAGC, establishing a robust foundation for the future development of perioperative treatment paradigms in gastric cancer and offering renewed hope for long-term survival benefits in LAGC patients.

Some limitations need to be declared in this study. Firstly, this was a retrospective study with inherent limitations in data collection, particularly regarding preoperative molecular biomarkers. Further studies should focus on the potential predictive value of the preoperative pathological indicators such as PD-L1 expression (CPS score), tumor mutation burden (TMB), MSI status, and multi-omics indicators, etc. Secondly, we mainly present the prediction of pathological response rather than the long-term prognosis because of the limited follow-up time. Thirdly, we didn’t unit the NICT regimens and cycles due to the small sample size. Fourthly, an independent external validation set need to be conducted to further explore the predictive ability of this nomogram. Despite these limitations, this nomogram represents a valuable step towards personalized prediction of treatment response in gastric cancer. Large-scale prospective studies should be conducted to provide high-level evidence in predicting the special population with better tumor response and survival benefits in the future.

## 5 Conclusions

LAGC patients who accepted NICT acquired superior tumor response with 16.2% of pCR. Complete response by abdominal enhanced CT, less diameter of tumor bed, non-signet-ring cell carcinoma, ages≥70 years old, and CEA<4.25 ng/mL were proved as the independent predictors for pCR. Nomogram model based on the above indicators could provide satisfactory predictive effect for the pCR in LAGC patients with NICT, which prove to be a valuable approach for surgeons to make personalized strategies.

## Data Availability

The raw data supporting the conclusions of this article will be made available by the authors on reasonable request, without undue reservation.
