# Supplementary material for: Development and validation of nomogram for predicting pathological complete response to neoadjuvant chemotherapy and immunotherapy for locally advanced gastric cancer: a multicenter real-world study in China
Source: Front Immunol. 2025 Jul 18;16:1603196. doi: 10.3389/fimmu.2025.1603196 (PMC12313473; doi:10.3389/fimmu.2025.1603196)

Supplementary Material

Development and validation of nomogram for predicting pathological complete response to neoadjuvant chemotherapy and immunotherapy for locally advanced gastric cancer: A multicenter real-world study in China

**Hao Cui^1,2^****^†^, Rui Li^1,2†^, Liqiang Song^3†^, Yongpu Yang^4†^, Zhen Yuan^1,2^, Xin Zhou^5^, Junfeng Du^8^, Chaojun Zhang^7^, Hong Xu^6^, Lin Chen^1,3^, Yan Shi^4^, Jianxin Cui^2^*, Bo Wei^1,2^***

*** Correspondence:**Bo Wei E-mail: weibo@301hospital.com.cn
Jianxin Cui Email: cuijx_doctor@163.com

# Supplementary Tables

**Supplementary Table 1. Characteristics of 74 pCR patients in the training and validation set**

| **No.** | **Sex** | **Age** | **cTNM stage** | **Signet-ring cell** | **Radiological response** | **CEA level** | **Tumor location** | **Diameter of tumor bed (cm)** |
| --- | --- | --- | --- | --- | --- | --- | --- | --- |
| **1** | Female | 84 | 3 | No | PR | 4.1 | Proximal | 2 |
| **2** | Male | 75 | 3 | No | PR | 1.5 | Proximal | 2.5 |
| **3** | Male | 75 | 3 | No | PR | 7.28 | Proximal | 1.5 |
| **4** | Male | 73 | 2 | No | CR | 1.99 | Distal | 0.5 |
| **5** | Female | 72 | 3 | No | CR | 2.42 | Distal | 2 |
| **6** | Male | 71 | 3 | No | CR | 2.71 | Distal | 3 |
| **7** | Male | 71 | 2 | No | PR | 3.77 | Middle | 1 |
| **8** | Male | 71 | 2 | No | CR | 2.72 | Distal | 0.8 |
| **9** | Male | 71 | 3 | No | PR | 1.71 | Proximal | 2 |
| **10** | Male | 71 | 3 | No | PR | 2.56 | Distal | 3 |
| **11** | Male | 71 | 3 | No | PR | 1.22 | Proximal | 2 |
| **12** | Female | 71 | 3 | No | PR | 3.9 | Proximal | 2 |
| **13** | Male | 70 | 3 | No | PR | 2.23 | Distal | 2.7 |
| **14** | Male | 69 | 3 | No | CR | 3.09 | Distal | 3 |
| **15** | Male | 69 | 3 | No | PR | 3.03 | Proximal | 2 |
| **16** | Male | 68 | 3 | No | PR | 4.68 | Proximal | 2 |
| **17** | Male | 68 | 3 | No | PR | 5.78 | Distal | 1.5 |
| **18** | Male | 68 | 2 | No | PR | 2.17 | Proximal | 3.5 |
| **19** | Male | 67 | 3 | No | PR | 4.81 | Distal | 1 |
| **20** | Male | 67 | 4a | No | PR | 59.35 | Proximal | 2 |
| **21** | Male | 67 | 3 | No | PR | 3.95 | Proximal | 1.2 |
| **22** | Male | 59 | 3 | No | CR | 3.29 | Distal | 1.5 |
| **23** | Male | 59 | 3 | No | PR | 1.86 | Distal | 1 |
| **24** | Male | 58 | 3 | No | PR | 3.61 | Proximal | 1.6 |
| **25** | Male | 57 | 3 | No | PR | 2.39 | Proximal | 3.5 |
| **26** | Male | 56 | 2 | No | PR | 3.02 | Distal | 2.5 |
| **27** | Male | 56 | 3 | No | PR | 1.39 | Distal | 2 |
| **28** | Male | 55 | 3 | No | PR | 2.16 | Middle | 2.3 |
| **29** | Female | 55 | 3 | No | PR | 1.41 | Proximal | 3.7 |
| **30** | Male | 52 | 3 | No | CR | 1.85 | Distal | 3 |
| **31** | Male | 51 | 3 | No | PR | 2.61 | Distal | 3.5 |
| **32** | Male | 50 | 3 | No | CR | 1.25 | Proximal | 1.5 |
| **33** | Male | 46 | 3 | No | CR | 4 | Proximal | 4.5 |
| **34** | Female | 32 | 2 | No | PR | 1.72 | Proximal | 1.5 |
| **35** | Male | 54 | 3 | No | PR | 1.93 | Proximal | 1.5 |
| **36** | Male | 55 | 3 | No | CR | 3.11 | Distal | 1.7 |
| **37** | Male | 70 | 3 | No | PR | 2.95 | Proximal | 4.5 |
| **38** | Male | 58 | 3 | Yes | SD | 3.05 | Middle | 7 |
| **39** | Male | 63 | 3 | No | PR | 2.25 | Proximal | 1 |
| **40** | Male | 66 | 3 | No | CR | 3.95 | Proximal | 4 |
| **41** | Male | 74 | 3 | No | PR | 4.25 | Distal | 3 |
| **42** | Male | 76 | 3 | No | PR | 2.63 | Middle | 3.5 |
| **43** | Male | 59 | 4a | No | CR | 3.73 | Distal | 1 |
| **44** | Male | 52 | 3 | No | CR | 1.85 | Distal | 3 |
| **45** | Male | 56 | 3 | No | PR | 0.22 | Distal | 0.3 |
| **46** | Male | 59 | 3 | No | CR | 2.58 | Middle | 3 |
| **47** | Male | 61 | 3 | No | PR | 2.36 | Proximal | 2 |
| **48** | Male | 62 | 3 | No | CR | 3.28 | Distal | 3 |
| **49** | Male | 50 | 3 | No | CR | 3.5 | Distal | 1.5 |
| **50** | Male | 44 | 3 | No | CR | 1.88 | Proximal | 2 |
| **51** | Male | 70 | 3 | No | CR | 4.3 | Proximal | 2.5 |
| **52** | Female | 64 | 3 | No | CR | 1.9 | Distal | 2.2 |
| **53** | Male | 53 | 3 | No | CR | 2.5 | Distal | 2 |
| **54** | Female | 60 | 3 | No | CR | 1.6 | Proximal | 1.8 |
| **55** | Female | 68 | 3 | No | CR | 0.9 | Proximal | 3 |
| **56** | Female | 51 | 3 | No | CR | 4.3 | Distal | 2 |
| **57** | Male | 61 | 3 | No | CR | 2.7 | Proximal | 4.5 |
| **58** | Male | 69 | 3 | No | CR | 1.01 | Proximal | 1.2 |
| **59** | Male | 54 | 3 | No | PR | 2.15 | Proximal | 7.6 |
| **60** | Male | 70 | 3 | No | CR | 2.15 | Proximal | 1.7 |
| **61** | Female | 71 | 2 | No | PR | 9.3 | Distal | 1.5 |
| **62** | Male | 69 | 3 | No | PR | 3.6 | Proximal | 0.5 |
| **63** | Female | 70 | 3 | No | PR | 2.8 | Distal | 2 |
| **64** | Male | 54 | 3 | No | PR | 0.6 | Distal | 2 |
| **65** | Male | 57 | 3 | No | PR | 4.1 | Proximal | 0.5 |
| **66** | Male | 62 | 3 | No | CR | 3.1 | Proximal | 0.7 |
| **67** | Male | 65 | 2 | No | PR | 2 | Proximal | 0.9 |
| **68** | Female | 53 | 2 | No | PR | 0.8 | Distal | 2 |
| **69** | Male | 66 | 3 | No | PR | 0.32 | Proximal | 3 |
| **70** | Male | 47 | 3 | No | PR | 1.21 | Middle | 2.5 |
| **71** | Female | 70 | 3 | No | PR | 1.08 | Proximal | 0.3 |
| **72** | Male | 60 | 2 | No | PR | 2.95 | Middle | 0.5 |
| **73** | Male | 66 | 3 | No | PR | 3.05 | Distal | 1.1 |
| **74** | Male | 58 | 3 | No | PR | 4 | Middle | 2.5 |

CEA, carcinoembryonic antigen.

# Supplementary Figures

**Supplementary figure 1. Subgroup analysis for nomogram based on clinical characteristics**

**(A)** Subgroup analysis for nomogram in all enrolled patients; **(B)** Subgroup analysis for nomogram in the training set; **(C)** Subgroup analysis for nomogram in the validation set.


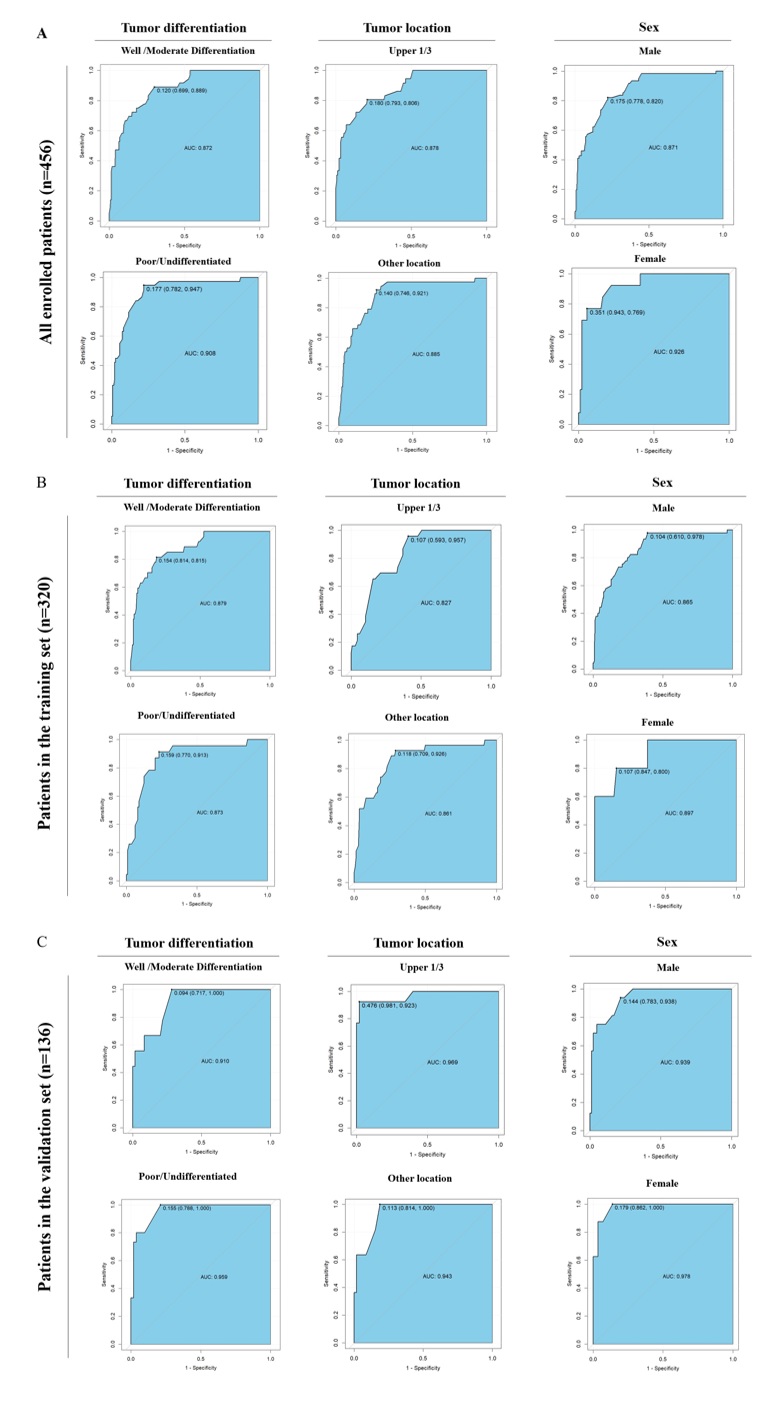


**Supplementary figure 2. ROC curve between nomogram and single variables for predicting pCR after NICT for LAGC patients**


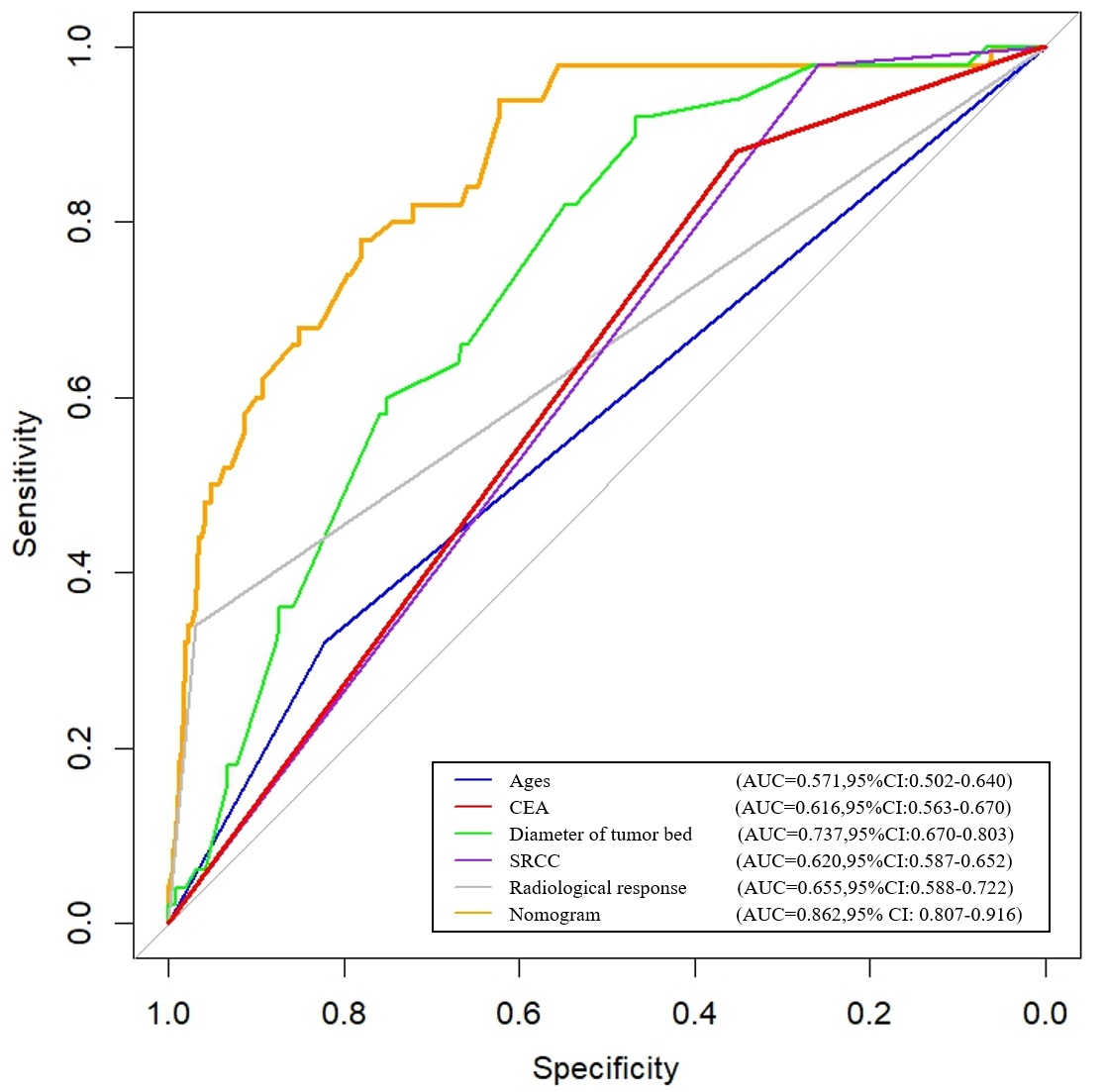

Supplement: Supplementary file 1 [file DataSheet1.docx]
